# Efficacy and safety of remimazolam besylate plus propofol for sedation in endoscopic retrograde cholangiopancreatography: a randomized controlled study

**DOI:** 10.3389/fmed.2026.1733994

**Published:** 2026-06-23

**Authors:** Changhui Deng, Xiaoxue Yu, Ting Yang, Jing Liao, Lin Zeng

**Affiliations:** 1 Department of Anesthesiology, Ziyang Central Hospital, Ziyang, China; 2 Department of Anesthesiology, Shifang People’s Hospital, Shifang, China

**Keywords:** a randomized controlled study, endoscopic retrograde cholangiopancreatography (ERCP), propofol, remimazolam, sedation

## Abstract

**Objective:**

To explore the efficacy and safety of the new anesthetic remimazolam besylate combined with propofol in anesthesia for endoscopic retrograde cholangiopancreatography (ERCP).

**Methods:**

A total of 60 patients who underwent selective ERCP in Shifang People’s Hospital were selected as the research objects. They were randomly divided into the control group (C group) and the remimazolam group (R group), with 30 cases in each group. C group was given normal saline + propofol, and R group was given remimazolam + propofol. The hemodynamic indexes, propofol dosage, sedation and analgesia effects, patient and doctor satisfaction, success rate of operation and perioperative complications of the two groups were compared.

**Results:**

The perioperative heart rate (HR) and mean arterial pressure (MAP) of R group were more stable than those of C group (*P* < 0.05) throughout the procedure. The dosage of propofol in R group was significantly less than that in C group (366.68 ± 69.22 mg vs. 436.34 ± 171.28 mg, *P* < 0.05). At each time point, the mean RSS score was higher in group R and the mean VAS score was lower (RSS: 5.03 ± 0.84 vs. 3.95 ± 0.81; VAS: 1.63 ± 1.18 vs. 2.63 ± 1.15, *P* < 0.05). The doctor satisfaction score of R group was significantly higher than that of C group (*P* < 0.05), while there was no significant difference in patient satisfaction score between the two groups (*P* > 0.05). There was no significant difference in the success rate of operation between the two groups (*P* > 0.05). The total incidence of perioperative complications in R group was significantly lower than that in C group (*P* < 0.05).

**Conclusion:**

The application of remimazolam besylate combined with propofol in ERCP can reduce the dosage of propofol, maintain stable hemodynamics, improve sedation and analgesia effects, enhance patient and doctor satisfaction, and reduce the incidence of perioperative complications, which is an effective and safe anesthetic scheme.

**Clinical trial registration:**

https://clinicaltrials.gov/, identifier ChiCTR2300 073982.

## 1 Introduction

Endoscopic retrograde cholangiopancreatography (ERCP) is an advanced endoscopic procedure widely used in the diagnosis and treatment of biliary and pancreatic diseases, such as gallstone extraction and stent placement (1). However, ERCP is an uncomfortable and painful procedure for patients. Compared with conventional gastrointestinal endoscopy, it requires deeper sedation and less body movement (2). Therefore, the choice of an appropriate anesthetic scheme is crucial to ensure the smooth progress of the operation and the safety of patients.

At present, a variety of sedative drugs with different anesthetic mechanisms have been used in ERCP. The combination of propofol and analgesics is the preferred sedative-analgesic method (3). Propofol is a short-acting intravenous anesthetic that can induce sleep quickly and allow patients to recover rapidly within 4–6 min (4). However, the increase in propofol dosage in deep sedation is usually accompanied by an increase in cardiopulmonary complications, such as respiratory depression, hypoxemia and hypotension, which may cause more perioperative problems especially in elderly patients undergoing anesthesia in the prone position (5, 6).

Remimazolam is a new type of anesthetic improved on the basis of midazolam. It makes up for the poor sedative effect of midazolam in clinical anesthesia (7). Studies have found that remimazolam can achieve sedative and analgesic effects similar to propofol. In the combined use group, the sedation score, pain score and pain mediators of patients were significantly improved or reduced, indicating that remimazolam can enhance the sedative and analgesic effects (8). Another study suggested that remimazolam combined with etomidate can improve the stress response of patients undergoing gastroscopy and colonoscopy. The mechanism is that remimazolam can exert an anti-inflammatory effect and effectively reduce the level of inflammatory factors in patients after operation (9). From the perspective of pharmacological properties, as one of the central nervous system drugs, benzodiazepines (including remimazolam) can block the neurotransmitters passing through the anesthetic nerves, reduce the secretion and expression of pain mediators such as PGE2 and IL-17, and then make the pain mediators act on the peripheral nerve endings to effectively relieve pain (10). In addition, remimazolam, as a new type of ultra-short-acting benzodiazepine anesthetic, has the characteristics of predictable sedation time and rapid recovery under painless gastroscopy (11). In our previous study, it was found that the combined use of remimazolam can reduce the dosage of propofol in painless gastroscopy, and has good efficacy and safety (12). However, there are few reports on the application of remimazolam in ERCP at present. Therefore, this study intends to explore the efficacy and safety of remimazolam besylate combined with propofol in ERCP anesthesia, so as to provide a better anesthetic scheme for perioperative anesthesia of ERCP.

## 2 Materials and methods

### 2.1 General Information

**FIGURE 1:**
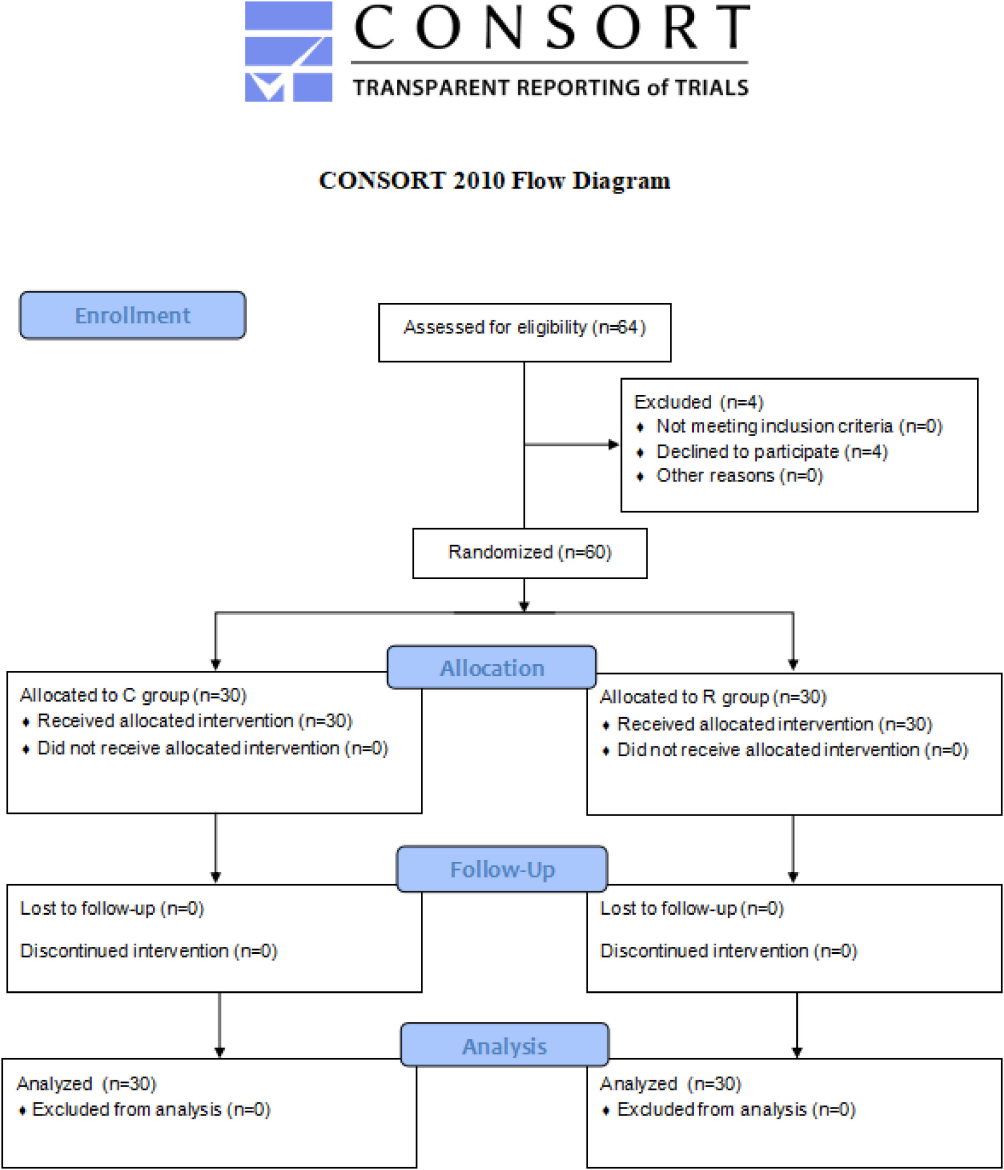
Consort diagram. Initially, 64 patients were randomly assigned to 1 of 2 groups as follows: the control group (C group) or the remimazolam group (R group). 60 patients (30 in C group and 30 in R group) completed this study.

A total of 64 patients who underwent selective ERCP in Shifang People’s Hospital from January 2024 to September 2025 were selected. After 4 refused to participate, 60 eligible participants were included in the final analysis. They were randomly divided into the control group (C group) and the remimazolam group (R group) (Figure 1). The inclusion criteria were as follows: (1) Scheduled for ERCP; (2) Aged 40–85 years; (3) American Society of Anesthesiologists (ASA) classification of I–III; (4) Body mass index (BMI) of 18–28 kg/m^2^. The exclusion criteria were: (1) Severe hypertension or diabetes; (2) Coronary heart disease; (3) Complicated with central nervous system diseases; (4) Difficult airway; (5) Allergic to the study drugs; (6) Intermittent or long-term use of benzodiazepines or opioids within 2 months before admission. This study was approved by the Ethics Committee of Shifang People’s Hospital (202306), and all patients and their families signed the informed consent. The study was registered in the Chinese Clinical Trial Registry (ChiCTR2300073982) on July 28, 2023.

### 2.2 Grouping method

A non-stratified random sequence was generated by a computer, and sealed in sequentially numbered envelopes to hide the random assignment. An anesthetic nurse who did not participate in the study was responsible for unified management. Before anesthesia, the anesthetic nurse opened the envelope to show the treatment assignment and prepared remimazolam or normal saline placebo. The same 50-mL syringe was used to extract the drug. Remimazolam was diluted with 0.9% normal saline to 25 mL (final concentration: remimazolam 1 mg/mL) or 25 mL of 0.9% normal saline was extracted. Before the completion of the final statistical analysis, the patients, surgeons, anesthesiologists and statisticians were all unaware of the assignment.

### 2.3 Anesthesia scheme

All patients fasted for 8 h and abstained from water for 2 h before the operation. When entering the operating room, they could take 10 ml of dyclonine hydrochloride mucilage orally and swallow it within 60 s. The patients were assisted to maintain the left lateral position, with their heads turned to the left. A thin pad could be placed on the chest and abdomen. A peripheral venous access was established in the upper limb, and compound sodium chloride injection was infused at 6 mL/kg⋅h. The changes in the patients’ signs were evaluated in detail.

In R group, 6 mg of remimazolam and 5 mg of oxycodone were administered intravenously, then 2–4 mg/kg of propofol was given for induction, and finally the anesthesia was maintained with 4–6 mg/kg⋅h of propofol. ERCP was performed when the RSS score reached 4–5. Drugs such as dopamine and atropine were used reasonably according to the signs to ensure the stability of vital signs. After the patient woke up from anesthesia, they were observed for 30–45 min, and transferred back to the ward if no abnormality was found. Flumazenil, a specific antagonist for remimazolam, was readily available in the operating room during the entire procedure for emergency reversal if necessary.

C group received the same anesthesia protocol except that remimazolam was replaced with the same volume of normal saline while all other medications and procedures were identical.

### 2.4 Observation indicators

#### 2.4.1 Hemodynamic indexes

The mean arterial pressure (MAP) and heart rate (HR) of the two groups were measured before drug administration (T1), before ERCP operation after drug administration (T2), at the start of ERCP (T3), 15 min after the start (T4) and at the end of ERCP (T5).

#### 2.4.2 Sedation and analgesia effects

At T1, T2, T3, T4, and T5 time points, the Ramsay Sedation Scale (RSS) and Visual Analog Scale (VAS) were used to score the sedation and analgesia of the two groups. The RSS score ranges from 1 to 6 points: 1 point means anxious and restless; 2 points means cooperative, oriented and quiet; 3 points means only responding to commands; 4 points means falling asleep, and responding quickly to light stimulation or calling; 5 points means falling asleep, and responding slowly to light stimulation or calling; 6 points means falling asleep, and no response to light stimulation or calling. The higher the score, the better the sedation effect. The VAS score ranges from 0 to 10 points: 0 points means no pain; 10 points means the most severe pain. The lower the score, the better the analgesic effect.

#### 2.4.3 Propofol dosage, recovery, and satisfaction

The total dosage of propofol used in the two groups during the operation was recorded. Thirty minutes after the operation, patient satisfaction and doctor satisfaction were evaluated using a 0–10 scale (0 = very poor, 10 = very good). Patient satisfaction was scored by the patients themselves according to intraoperative pain, discomfort, and overall perioperative experience. Doctor satisfaction was scored by the operating endoscopist according to sedation adequacy, patient body movement, and overall procedural conditions. The patient’s Steward awakening score was also investigated. The Steward awakening score ranges from 0 to 8 points: the higher the score, the fuller the recovery. A score of ≥ 6 points indicates that the patient can be transferred out of the recovery room.

#### 2.4.4 Operation success rate and perioperative complications

Operation success was defined as successful completion of ERCP with intended procedural interventions (e.g., diagnostic imaging, stone extraction, or stent placement) without unexpected termination due to sedation-related adverse events or technical failure. The success rate of ERCP in the two groups was recorded. The perioperative complications: respiratory depression (respiratory rate less than 12 times per minute, or no spontaneous breathing for more than 15 s), hypoxemia (arterial oxygen saturation less than 90%), auxiliary ventilation, choking cough, and nausea and vomiting were observed and recorded.

### 2.5 Sample size justification and statistical methods

In the preliminary research, the mean VAS score in R group at T2 was 1.3 (SD = 1.16), whereas the mean in C group was 2.2 (SD = 1.14). A two-samples *t-*test showed that the difference was no statistical significance for the time being, *P* = 0.097, Cohen’s *d* = −0.7826. According to the preliminary research results, the sample size calculation was based on the VAS scores at T2 as the primary outcome. Set α = 0.05 and power = 0.8 was used to determine the study. According to the formula estimate the effective sample size N ≈ 26 for each group. Invasive clinical trials often need to recruit more subjects to prevent patients from falling out during follow-up. Based on experience, it is assumed that 10% of the trials are lost to follow-up, so each group of samples is adjusted up to N* = N÷(1–0.1) ≈ 29.

Data analysis was achieved by using SPSS for Windows version 26.0. Normally distributed measurement data were expressed as mean ± standard deviation ($\bar{x}$ ± s). Continuous variables that were normally distributed were analyzed using an independent *t*-test, while non-normally distributed variables were analyzed using the Mann–Whitney U test for comparison between the groups. Repeated measures analysis of variance will be used for intra-group comparisons. The χ^2^-test were used for the comparison of count data. *P* < 0.05, indicating that the difference is statistically significant.

## 3 Results

### 3.1 General information

There was no significant difference in age, gender, BMI, ASA classification, complications and duration of procedure between the two groups (*P* > 0.05) (Table 1).

**TABLE 1:** Demographic data in the study groups.

Index	C group (*n* = 30)	R group (*n* = 30)	χ^2^/*t*	*P-*value
Age (years)	65.2 ± 14.26	61.03 ± 13.11	1.179	0.243
Body mass index (kg/m^2^)	22.45 ± 3.12	22.89 ± 3.17	−0.542	0.59
Gender (Male/Female)	12 月18 日	12 月18 日	2.4	0.121
ASA (II/III)	16/14	17/13	0.067	0.795
Duration of procedure (min)	65.43 ± 31.73	59.3 ± 26.22	0.758	0.452
Diabetes mellitus [n (%)]	5(16.7%)	4(13.3%)	0.131	0.718
Hypertension [n (%)]	13(43.3%)	12(40%)	0.069	0.793

ASA, American Society of Anesthesiology.

### 3.2 Hemodynamic indexes

From Table 2, both groups showed a significant decrease in HR during T2-T5 compared to T1 (*P* < 0.05). There was no statistically significant difference in HR between the two groups at T1 (*P* > 0.05). At T2-T5, HR in Group R was higher than that in Group C (*P* < 0.05). Compared with T1, the MAP in Group C significantly decreased during T2-T5 (*P* < 0.05). There was no statistically significant difference in MAP between the two groups at T1 (*P* > 0.05). MAP in Group R was higher than that in Group C at T2-T5 (*P* < 0.05).

**TABLE 2:** Comparison of hemodynamic indexes between the two groups.

Index	Time	C group (*n* = 30)	R group (*n* = 30)	*t*	*P-*value
HR (Bpm)	T1	85.27 ± 8.65	86.3 ± 7.76	−0.485	0.629
	T2	69.2 ± 10.07^#^	72.47 ± 6.33^*#^	−2.79	0.007
	T3	71.33 ± 11.49^#^	81.8 ± 8.26^*#^	−4.053	<0.001
	T4	69.2 ± 11.86^#^	80.73 ± 7.26^*#^	−4.541	<0.001
	T5	71.37 ± 12.72^#^	81.97 ± 7.87^*#^	−3.881	<0.001
MAP (mmHg)	T1	99.57 ± 10.53	97.1 ± 11.21	0.878	0.383
	T2	83.4 ± 9.71^#^	92.2 ± 10.94*	−3.294	0.002
	T3	87.11 ± 10.08^#^	94.26 ± 12.24*	−2.468	0.017
	T4	88.73 ± 10.18^#^	95. 68 ± 12.51*	−2.359	0.022
	T5	86.26 ± 8.8^#^	92 ± 10.69*	−2.272	0.027

HR, heart rate; MAP, mean arterial pressure; T1, before drug administration; T2, before ERCP operation after drug administration; T3, at the start of ERCP; T4, 15 min after the start of ERCP; T5, at the end of ERCP.

*P < 0.05 vs. control;

^#^*P* < 0.05 vs. T1.

### 3.3 Sedation and analgesia effects

At T1 time point, there was no significant difference in RSS score and VAS score between the two groups (*P* > 0.05). At T2, T3, T4, and T5 time points, the RSS score of R group was significantly higher than that of C group, and the VAS score was significantly lower than that of C group (*P* < 0.05) (Table 3).

**TABLE 3:** Comparison of sedation and analgesia effects between the two groups.

Index	Time	C group (*n* = 30)	R group (*n* = 30))	*t*	*P-*value
RSS score	T1	1.47 ± 0.51	1.53 ± 0.51	−0.456	0.65
	T2	4 ± 0.79^#^	5.03 ± 0.81^*#^	−4.986	<0.001
	T3	4 ± 0.87^#^	5.2 ± 0.85^*#^	−5.404	<0.001
	T4	3.83 ± 0.75^#^	4.93 ± 0.87^*#^	−5.245	<0.001
	T5	3.93 ± 0.87^#^	4.97 ± 0.85^*#^	−4.683	<0.001
VAS score	T1	3.43 ± 0.77	3.27 ± 0.74	0.821	0.415
	T2	2.73 ± 1.23^#^	1.37 ± 1.16^*#^	4.406	<0.001
	T3	2.67 ± 1.15^#^	1.67 ± 1.15^*#^	3.368	0.001
	T4	2.6 ± 1.16^#^	1.6 ± 1.22^*#^	3.254	0.002
	T5	2.63 ± 1.07^#^	1.87 ± 1.17^*#^	2.625	0.011

RSS, Ramsay Sedation Scale; VAS, Visual Analog Scale; T1, before drug administration; T2, before ERCP operation after drug administration; T3, at the start of ERCP; T4, 15 min after the start of ERCP; T5, at the end of ERCP.

*P < 0.05 vs. control;

^#^*P* < 0.05 vs. T1.

### 3.4 Propofol dosage, recovery, and satisfaction

The dosage of propofol in R group was significantly less than that in C group (*P* < 0.05). Thirty minutes after the operation, the doctor satisfaction score in R group was significantly higher than that in C group (*P* < 0.05), and there was no significant difference in patient satisfaction score between the two groups (*P* > 0.05). The awakening time of C group was longer than that of R group (*P* < 0.05) (Table 4).

**TABLE 4:** Comparison of propofol dosage, recovery and satisfaction between the two groups.

Index	C group (*n* = 30)	R group (*n* = 30)	*t*	*P-*value
Dosage of propofol (mg)	436.34 ± 171.28	366.68 ± 69.22*	2.065	0.043
Patient satisfaction score	9.07 ± 0.87	8.73 ± 0.87	0.903	0.37
Doctor satisfaction score	7.4 ± 1.57	8.3 ± 1.12*	−2.556	0.013
Steward awakening score	6.73 ± 0.83	7 ± 0.91	−1.201	0.235
Awakening time (min)	7.2 ± 2.5	5.93 ± 2.15*	2.11	0.039

*P < 0.05 vs. control.

### 3.5 Operation success rate and perioperative complications

There was no significant difference in the success rate of ERCP between the two groups (*P* > 0.05). There was no difference in the incidence of single complications between the two groups (*P* > 0.05), but the total number of complications in R group was lower than that in C group (*P* < 0.05) (Table 5).

**TABLE 5:** Comparison of operation success rate and total perioperative complications between the two groups.

Index	C group (*n* = 30)	R group (*n* = 30)	χ^2^	*P-*value
Operation success rate [n (%)]	28 (93.3%)	29 (96.7%)	0.345	0.557
Total perioperative complications [n (%)]	15 (50%)	7 (23.3%)*	4.32	0.038
Respiratory depression [n (%)]	5 (16.7%)	1 (3.3%)	3.214	0.073
Hypoxemia [n (%)]	3 (10%)	1 (3.3%)	1.059	0.303
Auxiliary ventilation [n (%)]	3(10%)	1(3.3%)	1.059	0.303
Nausea and vomiting [n (%)]	1(3.3%)	2(6.7%)	0.351	0.554
Choking cough [n (%)]	3(10%)	2(6.7%)	0.218	0.64

Total perioperative complications included individual adverse events: respiratory depression, hypoxemia, auxiliary ventilation, nausea and vomiting, and restlessness. Data are presented as n (%). *P < 0.05 vs. C group.

## 4 Discussion

ERCP is a minimally invasive procedure for the diagnosis and treatment of biliary and pancreatic diseases, but its technical complexity and long operation time often lead to obvious discomfort in patients, requiring effective sedation and analgesia (13). Propofol, as a commonly used intravenous anesthetic, has the advantages of rapid induction and recovery, but its narrow therapeutic window and dose-related cardiopulmonary depression limit its clinical application, especially in elderly or patients with underlying diseases (14). Therefore, exploring anesthetic regimens that can reduce propofol dosage while ensuring sedation and analgesia effects has become a research focus in the field of ERCP anesthesia.

The results showed that R group had more stable hemodynamic indexes at T2-T5 time points. This is consistent with the pharmacological characteristics of remimazolam: as a benzodiazepine receptor agonist, remimazolam has a moderate effect on the cardiovascular system, and its combined use with propofol can reduce the inhibitory effect of propofol on the sympathetic nervous system, thereby reducing fluctuations in blood pressure and heart rate (15). A previous study on painless gastroscopy also found that remimazolam combined with propofol can maintain more stable hemodynamics than propofol alone, which further supports our research results (16). Another reason may be that the dosage of propofol in Group R is lower, so the inhibition of cardiovascular system is reduced (17).

The sedation and analgesic effects are important indicators to evaluate the quality of anesthesia. In this study, the RSS score of R group was significantly higher than that of C group, and the VAS score was significantly lower than that of C group at T2-T5 time points. This indicates that remimazolam combined with propofol can not only improve the sedation effect but also enhance the analgesic effect. The analgesic effect of remimazolam may be related to its inhibition of the release of pain mediators such as prostaglandin E2 (PGE2) and interleukin-17 (IL-17) (10). In addition, remimazolam can reduce the stress response of patients during the operation, which also contributes to the improvement of analgesic effect (18).

In terms of propofol dosage, R group used significantly less propofol than C group. This might be because remimazolam has a synergistic sedative effect with propofol. Remimazolam can enhance the binding of propofol to γ-aminobutyric acid (GABA) receptors, thereby improving the sedative effect of propofol and reducing the required dosage of propofol (19). The reduction in propofol dosage is of great significance for reducing perioperative complications, as high-dose propofol is an important risk factor for respiratory depression and hypoxemia (20). In our study, the incidence of perioperative complications in R group was significantly lower than that in C group, which may be closely related to the reduction in propofol dosage.

Patient comfort and doctor satisfaction are important reflections of the clinical value of anesthetic regimens. R group had a higher doctor satisfaction score than C group, with no significant difference in patient satisfaction score between groups. The stable hemodynamics and less body movement of patients in R group created favorable conditions for the surgeon to operate, which improved doctor satisfaction (21). The lack of significant difference in patient satisfaction between the two groups may be attributed to the fact that patients in both groups achieved adequate moderate sedation during ERCP, and their overall comfort and perioperative experience were sufficiently satisfied. The awakening time of R group was shorter than that of C group, indicating that remimazolam combined with propofol has no adverse effect on the recovery of patients after operation, which is consistent with the characteristics of remimazolam’s rapid metabolism and short half-life (22).

There are some limitations in this study. First, this is a single-center study with a small sample size, which may limit the generalization of the research results. Future multi-center, large-sample studies are needed to further verify the conclusions. Second, the study included patients aged 40–85 years but did not perform subgroup analyses for elderly individuals or sex, both of which are associated with distinct differences in drug metabolism, sedative response, and safety profiles. Future studies with subgroup analyses stratified by age and sex are needed to confirm the generalizability of the results. Third, although flumazenil was available for emergency reversal, no patients required its use; the safety profile related to excessive sedation and reversal management warrants further observation in a larger population. Fourth, a remimazolam-only group was not included, and future studies are needed to explore its independent sedative effect.

## 5 Conclusion

In conclusion, the application of remimazolam besylate combined with propofol, compared to propofol alone, in ERCP anesthesia can reduce the dosage of propofol, maintain stable hemodynamics, improve sedation and analgesia effects, enhance doctor satisfaction, and reduce the incidence of perioperative complications.

## Data Availability

The original contributions presented in this study are included in this article/supplementary material, further inquiries can be directed to the corresponding author.
